# Evaluating dragging vs. point-by-point ablation strategies in cavotricuspidal isthmus ablation for atrial flutter: a retrospective single-center trial

**DOI:** 10.3389/fcvm.2025.1684646

**Published:** 2025-12-04

**Authors:** Marton Turcsan, Nina Kozima Kiraly, Kristof-Ferenc Janosi, Dorottya Debreceni, Botond Bocz, Dalma Torma, Peter Kupo

**Affiliations:** 1 Gottsegen National Cardiovascular Center, Budapest, Hungary; 2 Heart Institute, Medical School, University of Pecs, Pecs, Hungary

**Keywords:** cavotricuspidal isthmus, CTI ablation, atrial flutter, ablation, dragging ablation

## Abstract

**Background:**

Cavotricuspid isthmus (CTI) ablation is the preferred treatment for typical atrial flutter, performed using various techniques. This study aimed to compare procedural and follow-up data between point-by-point and continuous “dragging” radiofrequency (RF) catheter ablation methods.

**Methods:**

This retrospective, single-center study included 121 consecutive patients who underwent first-time RF CTI ablation for typical atrial flutter between January 2023 and August 2024. Patients were assigned to point-by-point (*n* = 49) or continuous dragging (*n* = 72) groups. All procedures were performed under conscious sedation using irrigated-tip catheters and intracardiac echocardiography. Patients with prior CTI ablation, cardiac surgery, or concomitant ablation were excluded. Procedural and follow-up outcomes were compared between groups.

**Results:**

The continuous dragging technique significantly shortened the time from the first to last ablation [12 (6; 27) min vs. 18 (11; 32) min; *p* < 0.05] and the time from the first ablation to the first CTI block [9 (8; 17) min vs. 13 (8; 25) min; *p* < 0.01]. Additionally, total ablation time [484 (285; 774) s vs. 704 (449; 955) s; *p* < 0.01] and energy usage [20,613 (11,191.5; 33,257.3) J vs. 25,717 (17,251.8; 36,420) J; *p* < 0.05] were lower in the dragging group. The dragging technique also increased the first pass block rate (69.4% vs. 46.2%; *p* < 0.01). There was no significant difference in overall procedure time [55 (46; 66) min vs. 58.5 (45; 72) min; *p* = 0.46], fluoroscopy duration (41 ± 6 s vs. 55 ± 8 s; *p* = 0.14), or acute reconnection rate (27.8% vs. 30.8%; *p* = 0.80). Both groups achieved a 100% acute success rate with no major complications. There was no significant difference in the rate of recurrence between the two groups (2.77% vs. 2.04%, *p* = 1.0) during the long-term follow-up (13.4 ± 3.8 months).

**Conclusion:**

The continuous “dragging” RF ablation technique for CTI ablation in typical atrial flutter enhances procedural outcomes compared to the point-by-point method, demonstrating reduced ablation time, lower energy consumption, and a higher first pass block rate, all without compromising efficacy or safety.

## 1 Introduction

Typical atrial flutter (AFl) is a prevalent type of supraventricular tachycardia characterized by a circular pathway that involves the cavotricuspidal isthmus (CTI), a narrow portion of tissue connecting the tricuspid valve and the inferior vena cava (IVC) (1). As outlined in the 2019 European Society of Cardiology (ESC) guidelines for managing supraventricular tachycardias, the recommended first-line approach for patients experiencing recurrent and symptomatic CTI-dependent atrial flutter is radiofrequency (RF) catheter ablation (2).

**Figure 1:**
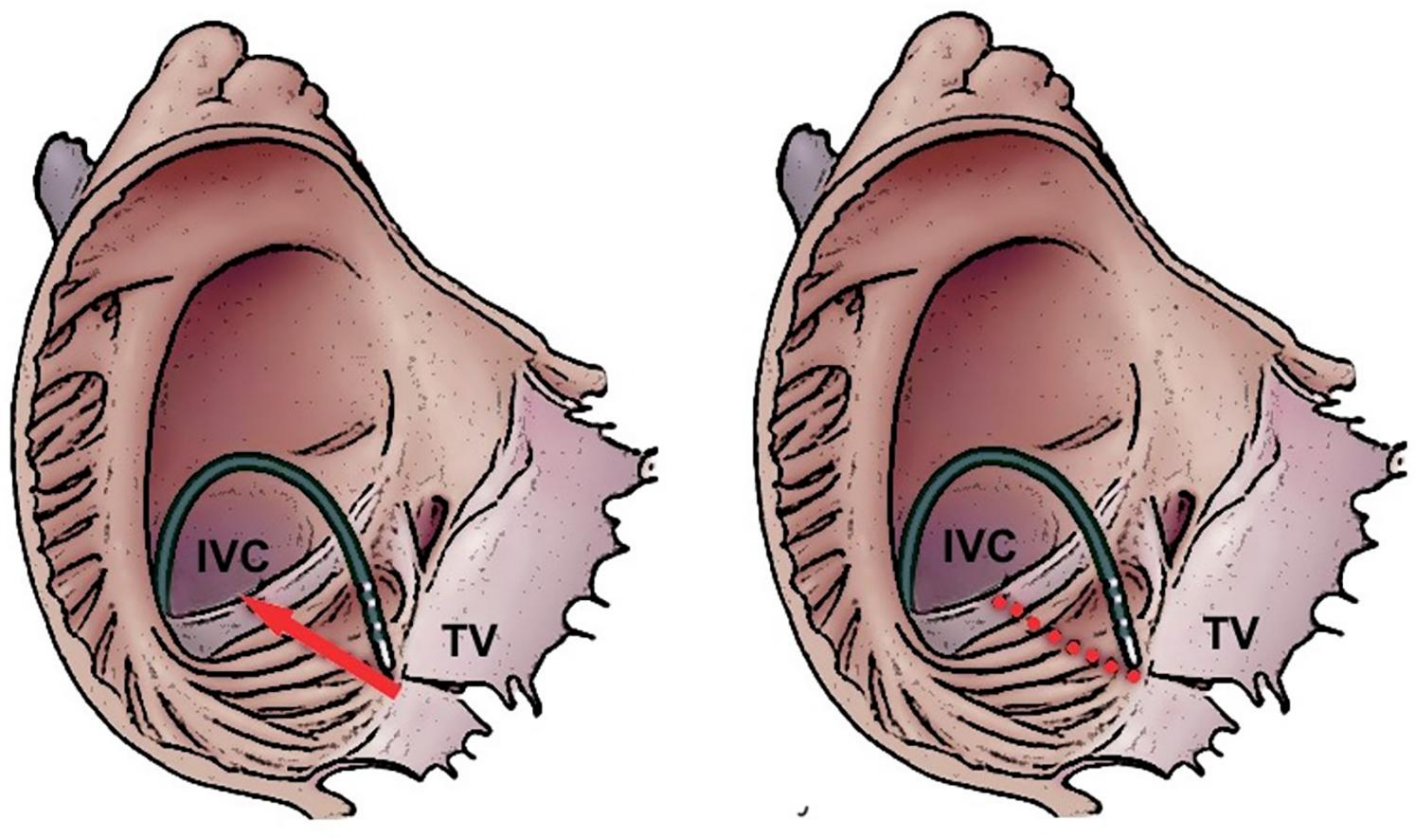
Dragging (left panel) and point-by-point (right panel) ablation strategies to creation an ablation line on the cavotricuspidal isthmus. IVC, inferior vena cava; TV, tricuspidal valve.

This procedure establishes bidirectional conduction block across the CTI and demonstrates a high degree of success in both short-term and long-term outcomes, accompanied by a low complication rate (2). The process of forming the ablation line can occur via different techniques: the dragging technique involves continuous RF ablation without direct interruption from the tricuspid annulus (TA) to the IVC. Additionally, the CTI ablation line can be created by applying a series of point-by-point applications from the TA to the IVC (Figure 1).

Previously, a randomized trial demonstrated that the dragging RF delivery technique has the potential to decrease procedure duration and fluoroscopy exposure, as well as reduce the total RF energy when compared with the point-by-point RF ablation technique in patients undergoing CTI ablation (3). In this study, an 8 mm non-irrigated ablation catheter was utilized. To date, there is no scientific data comprising the two different ablation techniques with irrigated-tip catheters. Additionally, intracardiac ultrasound (ICE) was not used in the previous study, a tool that has been demonstrated to enhance procedural outcomes in cases of typical AFl ablations (4–6).

The aim of our study was to compare procedural outcomes between dragging and point-by-point ablation approaches in patients who underwent ICE-guided CTI ablation due to typical AFl.

## 2 Methods

### 2.1 Study population

In our retrospective, single-center trial, we included 121 patients who underwent RF CTI ablation for either ongoing or documented typical AFl at our university center between January 2023 and August 2024. Patients were divided into two groups according to the technique used for CTI ablation: Point-by-point group included patients who underwent CTI ablation using the point-by-point creation of the ablation line with short interruptions for a certain period of time between the ablations. With the dragging technique, the aim of the operator was to create the ablation line alongside the CTI from the TA to the IVC without any interruption. If it was still necessary (e.g., due to sudden catheter dislocation or entrapment), the ablation line was also continued using the point-by-point technique. We defined an approach as dragging, if the ablation stayed at one point for long enough time and moved to the next point without interruption of energy delivery (continuous approach).

Inclusion criteria were: (1) age ≥18 years, (2) ongoing or previously documented typical cavotricuspid isthmus–dependent atrial flutter, and (3) patients who underwent first-time CTI ablation using either dragging or point-by-point technique at our institution.

Exclusion criteria were: (1) prior CTI ablation or redo procedures, (2) history of cardiac surgery or previous left atrial ablation procedures, (3) concomitant ablations for other arrhythmias during the same procedure, and (4) mixed procedures where both ablation techniques were applied in the same patient.

The study protocol fulfilled with the principles outlined in the Declaration of Helsinki. Informed consent was obtained following approval from the ethics research board of our institution. All patients underwent a comprehensive baseline clinical evaluation, including review of medical history, electrocardiography, routine blood tests, and echocardiography.

### 2.2 CTI ablation procedure

All catheter ablation procedures were conducted by a single experienced operator, a board-certified electrophysiologist with over five years of expertise in the field. The electrophysiological study and ablation were performed under conscious sedation using midazolam and fentanyl, with patients having fasted and remaining on uninterrupted anticoagulation therapy. After administering local anesthesia and obtaining femoral venous access, a decapolar steerable catheter (Dynamic Deca, Bard Electrophysiology, Lowell, MA, USA) with an interelectrode spacing of 2–5–2 mm was advanced into the coronary sinus (CS). Concurrently, a 7F 4 mm irrigated-tip ablation catheter (Alcath Black Flux G, Biotronik, Berlin, Germany) was introduced into the right atrium. The procedures utilized an 8F intracardiac echocardiography (ICE) catheter (AcuNaV™ 90 cm, Siemens Medical Solutions, Mountain View, CA, USA), but electroanatomical mapping systems (EAMS) were not employed.

For cases where arrhythmias were ongoing, entrainment mapping was used to confirm cavotricuspid isthmus (CTI) dependence of the flutter. If the arrhythmia was successfully terminated by radiofrequency (RF) ablation, the procedure was concluded with CS stimulation. In patients with documented typical atrial flutter (AFl) but presenting in sinus rhythm at the time of the procedure, ablation was performed while maintaining continuous proximal CS pacing. Comprehensive data acquisition, including twelve-lead electrocardiograms and intracardiac electrograms, was systematically recorded and archived using a digital system (CardioLab, GE Healthcare, Chicago, IL, USA) with a band-pass filter set between 30 and 500 Hz to ensure high-fidelity signal processing.

The goal of RF ablation was to create a complete linear lesion along the CTI. The operator employed either a point-by-point or a dragging technique based on procedural discretion. Temperature-controlled ablation was maintained at a target of 43 °C, with power delivery restricted to 45 W and irrigation set at a constant flow of 15 mL/min. The procedural endpoint required the fulfillment of three key criteria: successful termination of the arrhythmia, confirmation of bidirectional isthmus block, and the establishment of a continuous conduction block. This block was identified by distinct local double potentials along the ablation line, with an isoelectric segment separating two sharp electrograms, signifying successful CTI modification. Bidirectional block was systematically verified by pacing from both the coronary sinus and the low lateral right atrium, combined with differential pacing to exclude residual slow conduction across the CTI.

### 2.3 Study endpoints

Acute success was established by confirming the persistence of bidirectional conduction block along the CTI following a 20-min waiting period after the last ablation. First-pass block was characterized by the presence of bidirectional conduction block along the CTI, either occurring prior to or immediately following completion of the CTI line ablation, and without the need for supplementary ablations. Procedure duration, quantified in minutes, defined as the interval from the initiation of the first femoral puncture to the withdrawal of the final venous sheath. Therapy duration, also measured in minutes, was defined as the duration between the first and last of RF application. The time from the first ablation to the first block was also measured in minutes. Fluoroscopy time, recorded in seconds, along with the corresponding radiation dose, was systematically documented by the fluoroscopy system. Furthermore, the duration of ablation, expressed in seconds, was accurately calculated and archived using the EP recording system. Major complications were defined as instances involving pericardial effusion/tamponade or vascular complications, which encompassed significant hematomas requiring intervention or prolonged hospitalization, arteriovenous fistulas, and pseudoaneurysms. Recurrence was defined as the reappearance of typical atrial flutter documented on surface ECG during long-term follow-up.

### 2.4 Statistical analysis

The distribution pattern of the data was assessed using Shapiro–Wilk tests, with all tests conducted as two-tailed tests at a significance level of *p* < 0.05. Continuous data were reported as either mean ± standard deviation (SD) or median (interquartile range, IQR), depending on suitability. Categorical variables were presented as absolute numbers and percentages. Chi-square test, *T*-test, and Mann–Whitney *U*-test were utilized for appropriate comparisons. Statistical analyses were conducted using SPSS 28 software (SPSS, Inc., Chicago, IL, USA).

## 3 Results

The study encompassed a total cohort of 121 patients. In this cohort, 72 patients underwent CTI ablation using the dragging technique (classified as the “Dragging group”), while the remaining 49 individuals were treated with the point-by-point method (referred to as the “Point-by-point group”). The study population included 93 male participants, representing 76.85% of the total cohort. Baseline characteristics were comparable between the two groups, with no statistically significant differences observed. A detailed summary of these baseline characteristics is provided in Table 1.

**Table 1:** Baseline characteristics of the study population. TIA, transient ischemic attack.

Variables	Dragging group (*n* = 72)	Point-by-point group (*n* = 49)	*p* Value
Age (years)	67.9 ± 8.9	70.0 ± 7.9	0.25
Male/Female	56/16	37/12	0.77
Hypertension (%)	63 (87.5%)	36 (73.4%)	0.08
Heart failure (%)	21 (29.2%)	14 (28.5%)	0.89
Coronary artery disease (%)	22 (30.5%)	10 (20.4%)	0.30
Diabetes mellitus (%)	34 (47.2%)	14 (28.5%)	0.06
Chronic kidney disease (%)	12 (16.6%)	2 (4%)	0.07
Atrial fibrillation (%)	26 (36.1%)	13 (26.5%)	0.36
Prior stroke/TIA (%)	8 (11.1%)	4 (8.1%)	0.82

**Figure 2:**
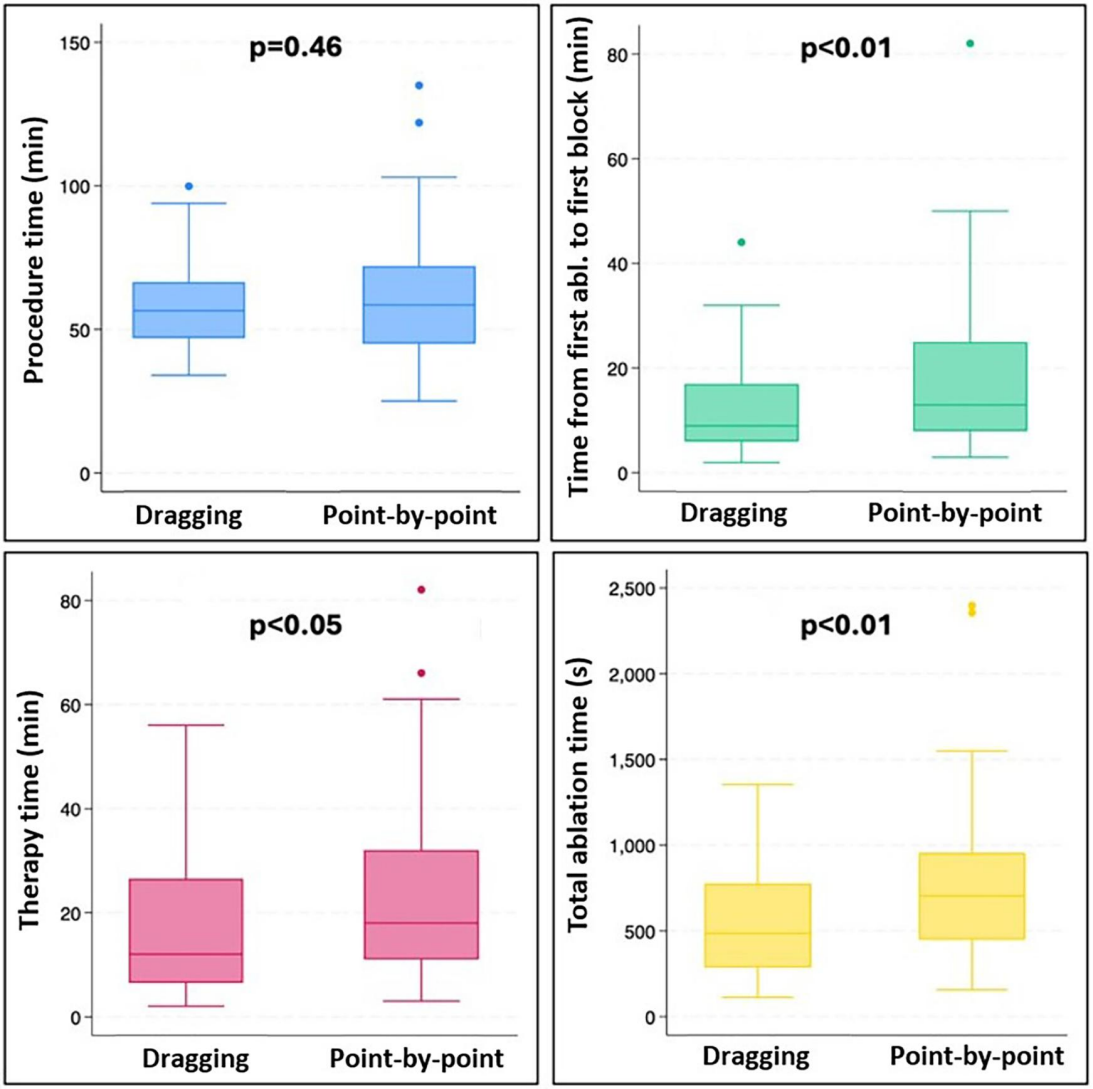
Box plots presenting procedure time (blue), time from fist ablation to first block (green), therapy time (pink), and total ablation time (yellow) in the dragging and the point-by-point group.

In each case, a successful bidirectional isthmus block was achieved after a waiting period of 20 min, resulting in a 100% acute success rate. The total procedure time did not differ between the groups [dragging group: 55 (46; 66) min vs. point-by-point group: 58.5 (45; 72) min, *p* = 0.46]. Notably, a significant reduction in total ablation time [484 (285; 774) s vs. 704 (449; 955) s, *p* < 0.01] and therapy duration [12 (6; 27) min vs. 18 (11; 32) min, *p* < 0.05] was observed in the dragging group compared to the point-by-point group. There was also a significant difference favoring the dragging group in the first pass block rate (69.4% vs. 46.2%, *p* < 0.01), time between the first ablation and the first block [9 (8; 17) min vs. 13 (8; 25) min, *p* = 0.01], and total ablation energy [20,613 (11,191.5; 33,257.3) J vs. 25,717 (17,251.8; 36,420) J, *p* < 0.049]. No significant difference was found between the two groups regarding total fluoroscopy time [0 (0; 85) s vs. 37 (0; 89) s, *p* = 0.26], and acute reconnection rate (27.8% vs. 30.8%, *p* = 0.80). However, the total fluoroscopy dose was reduced when applying the dragging technique [0.2 (0; 2.2) mGy vs. 0.6 (0.1; 4.6) mGy, *p* = 0.04]. No significant complications were observed within the study cohort. During the average follow-up of 13.4 ± 3.8 months, there was no significant difference in the rate of recurrence between the two groups (2.77% vs. 2.04%, *p* = 1.0). A summary of the results is shown in Table 2 and Figure 2.

**Table 2:** Procedural parameters in the study population.

Variables	Dragging group (*n* = 72)	Point-by-point group (*n* = 49)	*p* Value
Procedure time (min)	55 (46; 66)	58.5 (45; 72)	0.46
Therapy duration (min)	12 (6; 27)	18 (11; 32)	<0.05
Time from first ablation to first block (min)	9 (8; 17)	13 (8; 25)	<0.01
Fluoroscopy time (sec)	41 ± 6	55 ± 8	0.14
Fluoroscopy dose (mGy)	0.2 (0; 2.2)	0.6 (0.1; 4.6)	0.04
Total ablation time (sec)	484 (285; 774)	704 (449; 955)	<0.01
Total ablation energy (J)	20,613 (11,191.5; 33,257.3)	25,717 (17,251.8; 36,420)	<0.05
First pass block	69.4%	46.2%	<0.01
Acute reconnection	27.8%	30.8%	0.80
Acute success rate (%)	100	100	1.0
Major complications	0	0	NA
Recurrence rate (%)	2.77	2.04	1.0

## 4 Discussion

In this single-center retrospective study, we performed a comparative analysis of procedural and follow-up data between two groups: dragging and point-by-point CTI ablations. Our findings did not reveal any statistically significant differences in acute and long-term success rates, acute reconnection rates, fluoroscopy time, procedure time and complication rates between the groups. However, the dragging group demonstrated several notable advantages over the point-by-point group, including shorter total ablation time, therapy duration (time from first to last ablation), and time from first ablation to first block, with a significantly higher first pass block rate. Although ablation and energy delivery times were markedly shorter with the dragging technique, the overall procedure duration remained similar between the two groups. This can be attributed to several procedural steps—such as vascular access, catheter positioning, intracardiac echocardiography setup, and post-ablation validation of bidirectional block—that are independent of the ablation method. Therefore, the improvement in ablation efficiency does not necessarily translate into a shorter overall procedure time.

Catheter ablation plays a crucial role in the long-term treatment of AFl by aiding in maintaining sinus rhythm. The 2019 ESC guidelines for managing patients with supraventricular tachycardia recommend catheter ablation for people experiencing symptomatic and recurrent episodes of CTI-dependent flutter. Furthermore, the ESC guidelines suggest considering CTI ablation after the first symptomatic episode of typical AFl (2). The primary goal of CTI catheter ablation is to create a continuous lesion along the cavotricuspid isthmus, ultimately achieving bidirectional conduction block. This block signifies the complete cessation of electrical propagation across the CTI in both directions, effectively disrupting the reentry circuit responsible for typical atrial flutter. As a result, bidirectional block is widely accepted as the procedural endpoint for CTI ablation. Extensive research consistently highlights the high acute and long-term success rates of this procedure, with multiple studies reporting success rates exceeding 90% (2, 7–9).

The effectiveness of the CTI ablation relies on establishing continuous and transmural linear lesions. Incomplete tissue penetration or discontinuities within the lesions are commonly regarded as causes of procedural failure (10, 11). To achieve the necessary lesions for establishing bidirectional conduction block, the ablation catheter can be either continuously dragged along the CTI during RF energy application (dragging) or moved sequentially from one point to the next (point-by-point) with each energy application (3, 12).

To date, only one study has compared the two techniques in CTI ablations. This single-center, prospective randomized trial, published in 2007 and involving 40 patients, found significantly shorter fluoroscopy times in the dragging group. Consistent with our findings, the dragging approach required lower total ablation energy to achieve the procedural endpoint, had a shorter treatment duration (time from the start of RF delivery to the completion of CTI block), and demonstrated a significantly higher rate of first pass block. However, skin-to-skin procedure time was not reported (3). Two major differences between our study and the aforementioned one should be highlighted. Firstly, the previous study utilized a non-irrigated 8 mm catheter for CTI ablation, whereas we employed 4 mm irrigated-tip catheters in our comparison. Additionally, all procedures in our study were ICE-guided, allowing for direct, real-time visualization of anatomy during ablation.

Beside above, the procedural outcomes of CTI ablation are significantly influenced by various factors, including the type of ablation catheter used, the application of intracardiac ultrasound, and the energy settings during the procedure. Understanding these elements is crucial for optimizing the efficacy and safety of the ablation process. The choice of ablation catheter is a critical determinant of procedural success. Traditional non-irrigated catheters have been widely used, but recent advancements have introduced irrigated-tip catheters that allow for better cooling and larger lesion formation.

Several previous trials have compared irrigated-tip catheters to 8 mm non-irrigated catheters for CTI ablations and found no significant differences in procedural data (procedure time, total ablation energy, fluoroscopy time) between the groups (13–16). However, a study reported that using an 8-mm non-irrigated catheter at power settings of 70–100 W led to complications such as right coronary artery (RCA) damage, highlighting the importance of catheter selection and power settings in minimizing risks (17, 18). Currently, contact force (CF) sensing catheters are available for CTI ablations; however, a meta-analysis involving 761 patients from 10 studies showed that using CF-sensing catheters provided no additional benefits in terms of procedural time, fluoroscopy time, acute success, or complication rates compared to conventional ones (19).

The energy settings during ablation play a pivotal role in determining the size and quality of the lesions created. Higher power settings (e.g., 50–100 W) have been associated with increased lesion depth and efficacy, but they also carry a risk of collateral damage to surrounding structures. The lesion size index (LSI) has been proposed as a metric to correlate with successful outcomes, emphasizing the need for careful monitoring of contact force and energy delivery during the procedure (20).

ICE allows for safe, real-time visualization of intracardiac structures, aiding catheter manipulation, improving tissue-catheter contact, and facilitating early recognition of potential procedural complications. Numerous trials have demonstrated that ICE can enhance procedural outcomes in patients undergoing CTI ablation (4, 6, 21). In our current trial, all ablation procedures were ICE-guided, resulting in minimal fluoroscopy time and exposure during the procedures. Moreover, while using ICE can improve procedural outcomes and minimize required fluoroscopy, a total zero-fluoroscopy approach is also feasible even without EAMS guidance in CTI ablations, as recent papers have shown (5, 22).

In addition to fluoroscopy-only and ICE-guided techniques for CTI ablations, an EAMS-guided approach is available (23). Several trials have demonstrated that utilizing this strategy can achieve total elimination of required fluoroscopy (24–26). However, there is currently no available scientific data comparing dragging vs. point-by-point ablation techniques for EAMS-guided CTI ablations. Nonetheless, during EAMS-guided ablations, the virtual visualization of ablations on the 3D mapping is influenced by the starting and termination of RF delivery, potentially resulting in less precise visualization of ablation points on the map in cases of dragging ablation.

In addition to EAMS-guidance, recent publications have investigated the use of visualizable catheter and sheath systems in CTI ablations. These technologies enable full three-dimensional visualization of the catheter shaft and tip within the mapping system, allowing operators to perform complex maneuvers with greater control and accuracy. Studies have reported that incorporating such visualizable sheaths into CTI ablation workflows can significantly reduce total ablation time and total procedure time, while maintaining high acute success and low complication rates. Additionally, these approaches have been associated with higher first-pass bidirectional block rates and near-zero fluoroscopy exposure, even in anatomically challenging CTI regions (27, 28).

While our study focused on comparing dragging vs. point-by-point RF ablation in typical atrial flutter, Pulsed field ablation (PFA) represents an emerging technique that could further expand procedural options. It is a novel non-thermal energy modality that induces irreversible electroporation in cardiac tissue, offering a potential alternative to conventional RF ablation (29).

Several recent studies have investigated the use of PFA for typical atrial flutter ablation, using different catheter designs and energy delivery systems. A prospective, observational trial involving 311 patients reported high acute success rates and a favorable safety profile when applying PFA for CTI and atypical atrial flutter ablations, suggesting that this approach could be an efficient and safe alternative to conventional thermal energy (30). Another case study evaluated a balloon-in-basket PFA catheter, demonstrating successful CTI ablation with promising procedural outcomes, further confirming the feasibility of non-thermal lesion creation in this anatomical region (31). In contrast, a study utilizing a focal monopolar PFA catheter reported effective CTI ablation but noted potential challenges, including periprocedural coronary spasms and transient conduction disturbances, emphasizing the need for cautious evaluation of this new energy source (32).

Taken together, these findings indicate that PFA—applied through various catheter technologies—may provide a valuable addition to the therapeutic options for typical Afl. However, its long-term efficacy, optimal application parameters, and safety profile remain to be established through further dedicated studies. Nonetheless, conventional radiofrequency ablation remains a well-established and reliable approach, and the findings from our study offer important insights into optimizing RF techniques, reinforcing their central role in current clinical practice.

## 5 Limitations

Several limitations must be acknowledged. First, the retrospective nature of the study means that data collection and analysis were performed after the events had occurred, introducing potential risks of selection bias, missing data, and difficulties in controlling for confounding variables that may influence the results. Second, as this was a single-center study conducted at a high-volume electrophysiology center with standardized procedural protocols and experienced operators, the findings may not be fully generalizable to other institutions with different patient populations, procedural settings, or operator experience. These factors may limit the external validity of our results. Future multicenter, prospective trials with larger and more heterogeneous patient cohorts are warranted to confirm the reproducibility and broader applicability of our observations.

## 6 Conclusion

In our single-center retrospective trial, we found that the dragging approach in patients with AFl undergoing CTI ablation is beneficial compared to the point-by-point ablation technique. Utilizing the dragging technique, treatment time, total ablation time, and total RF delivery can be shortened with a higher first pass block rate without influencing the efficacy or safety of the procedure.

## Data Availability

The raw data supporting the conclusions of this article will be made available by the authors, without undue reservation.
